# Quasi-continuous X-ray generation in LiTaO_3_-based pyroelectric accelerator driven by periodically varying temperature

**DOI:** 10.1038/s41598-025-15436-4

**Published:** 2025-08-25

**Authors:** P. Karataev, M. Ali, K. Fedorov, A. Kubankin, V. Margaryan, A. Oleinik, A. Shchagin

**Affiliations:** 1 John Adams Institute at Royal Holloway, University of London, Egham, TW20 0EX Surrey UK; 2 Belgorod National Research University, Pobedy St. 85, Belgorod, 308015 Russia; 3 Institute of Applied Problems of Physics NAS RA, 25 Hr. Nersisyan Street, Yerevan, 0014 Armenia; 4 Deutsches Elektronen-Synchrotron DESY, Notkestr. 85, 22607 Hamburg, Germany; 5 Kharkov Institute of Physics and Technology, 1, Akademicheskaya St, Kharkov, Ukraine

**Keywords:** Ferroelectrics and multiferroics, Thermoelectric devices and materials, Thermoelectrics, Mass spectrometry

## Abstract

Nowadays, X-ray sources are advanced tools used to probe the properties of matter and dynamic systems. Large scale accelerator based light source facilities have been constructed around the Globe to meet the scientific, technological and societal needs. Development of compact pocket-style X-ray systems meeting safety regulations is a complementary task requiring stable, reliable, and reproducible sources of X-rays with controllable parameters. In this research we employed a new approach for pyroelectric accelerator operation. Periodically varying temperature of LiTaO_3_ crystal enabled us to generate stable quasi-continuous X-ray beam. We managed to observe and characterise the avalanche process at pyroelectric effect. This phenomenon is responsible for multiplication of electrons at the anode while producing X-rays. We have demonstrated the great potential of this compact X-ray source by performing reliable energy-dispersive X-ray spectroscopy for element analysis of two solid samples.

**Supplementary Information:**

The online version contains supplementary material available at 10.1038/s41598-025-15436-4.

Changing the temperature of Lithium Tantalate single crystal at moderate vacuum conditions of less than 10^−2^ Torr leads to generation of strong electric field^1–3^ with strength of about 10^5^– 10^6^ V/cm. This phenomenon is possible in LiTaO_3_ because of pyroelectric effect, appearing when the crystal is polarised due to applied temperature gradient^4–6^. A moderate vacuum is needed to avoid any shielding of the electric field by polarized air molecules, to prevent from current leak due to humid air, and to accumulate the charge at polar surfaces of the crystal^1^. Such a strong electric field is accompanied by field electron emission^7,8^ and impact ionization of the residual gas molecules^9^. The electrons and positive ions are accelerated in the electric field and interact with surroundings^1,10–16^. The electrons slow down in matter generating bremsstrahlung and characteristic X-ray photons. The characteristic X-ray lines allow to identify the sample material^17–40^which can directly be applied for its element analysis. If a grounded target is placed at a distance from the crystal, the electric field is locked onto it. If the crystal surface is positively charged, the electrons are ejected from the target, gain energy in the gap and generate X-rays from the crystal^22^ (Fig. 1b). When negative charge is accumulated at the crystal surface, the field emission results in ejection of electrons form the crystal, gaining energy in the gap, self-focusing, and generating X-rays from the target^10,13^ (Fig. 1c).

Pyroelectric sources are perspective and significantly broaden the potential of X-ray diagnostics^22,29,32,34,35,37–39^ including extreme conditions^39^. Compact generation scheme enables us to create a miniature, pocket style diagnostics device that can be brought to the investigated sample and provide an opportunity in scientific research which is complementary to large scale central facilities. Moreover, moderate X-ray energy, low power, and the absence of a high power external source generally satisfies safety requirements providing research and educational opportunities for small university or school group.

**Fig. 1:**
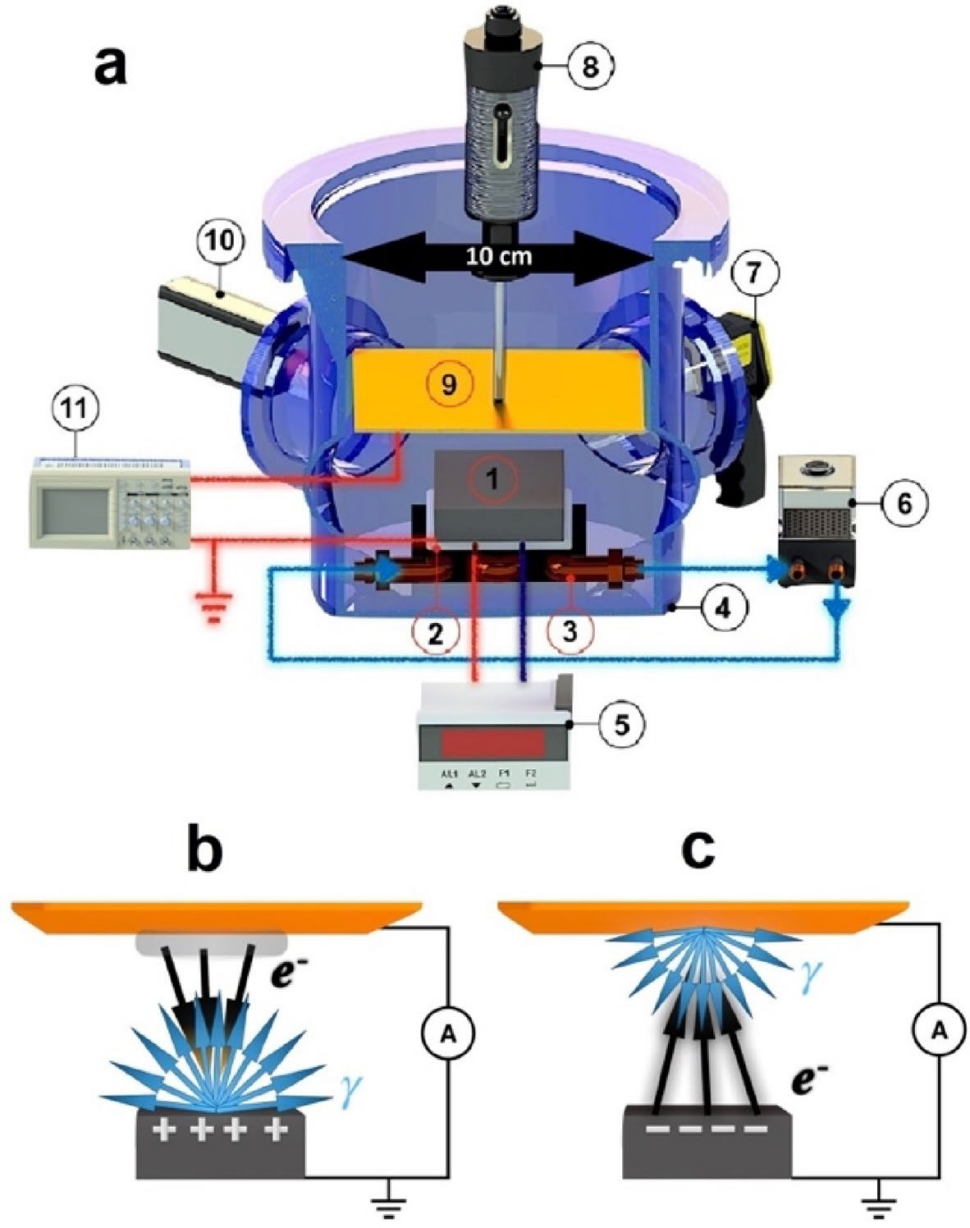
Schematic layout (**a**) of the experimental installation. (1) The assembly of LiTaO_3_ single crystal; (2) Peltier element; (3) radiator for water cooling; (4) vacuum chamber; (5) arbitrary waveform generator; (6) cooling water pump; (7) infra-red camera; (8) vacuum actuator; (9) target (brass or stainless steel); (10) semiconductor X-ray detector; (11) pico-ammeter. **(b)** Generation of electrons at positive polarity: the electrons are ejected from the target surface (yellow plate), gain energy in the gap, multiply the number ionizing the residual gas, and generate X-rays from the crystal (grey box); **(c)** Generation of electrons at negative polarity: the electrons are ejected from the surface of the crystal, gain energy in the gap, multiply the number ionizing residual gas, and generate X-rays from the target material. The operation principle is described in Methods.

The first step towards practical realization has been made by the Amptek company^40^. However, unstable X-ray emission, lack of reproducibility and repeatability of the X-ray spectra complicate the experimental result interpretation and limit the use of pyroelectric sources for practical applications. Insufficient understanding of the processes between the target and the crystal^15,18,22,24,26,36^ tightly linked to peculiarities of the charge formation at the crystal surface^13,22,31,41^ has become a fundamental challenge addressed in this paper.

**Fig. 1:**
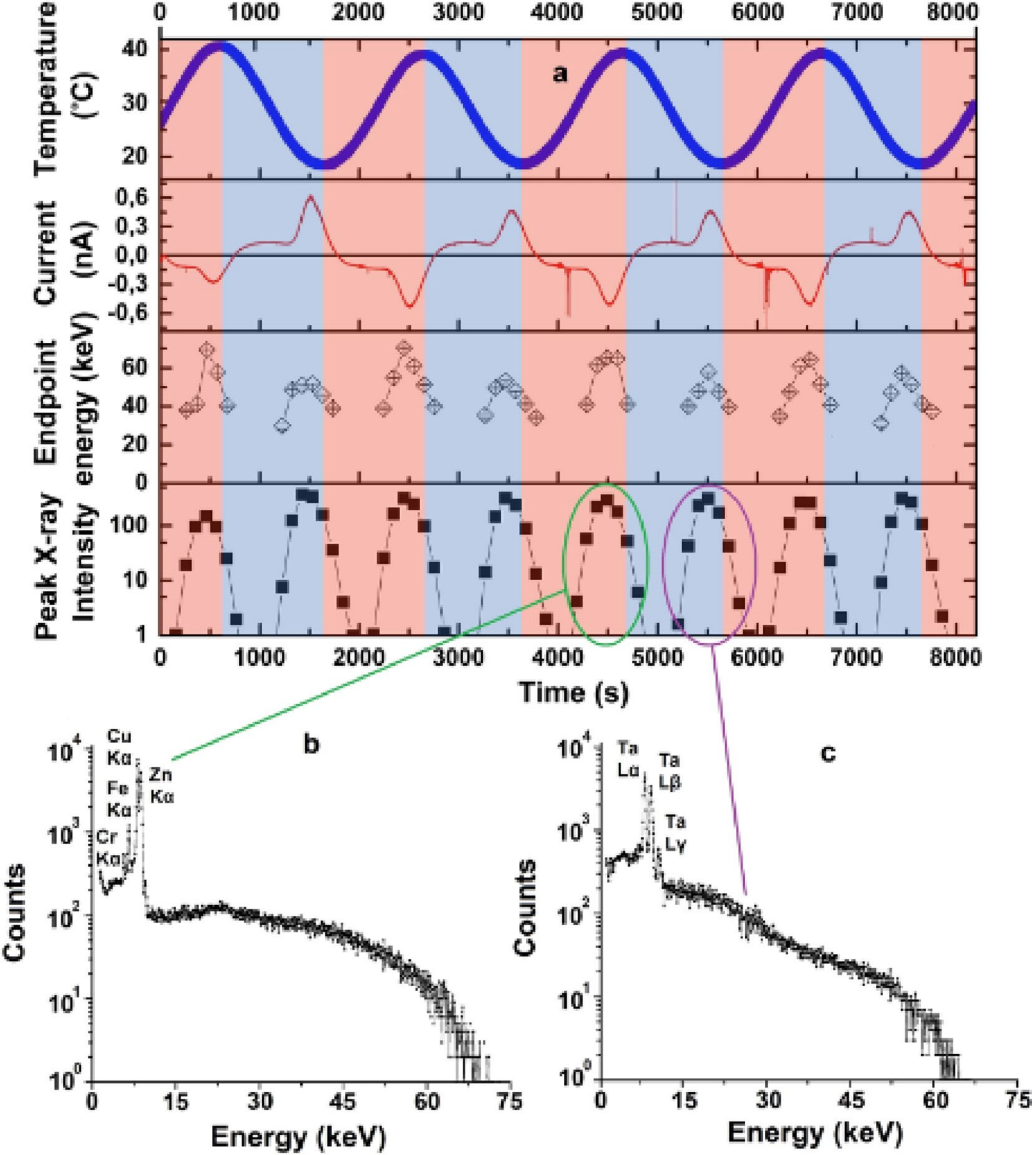
Results of the measurements for 0.5 mHz temperature variation frequency and 1.0–2.5 mTorr pressure range. **(a)** Four graphs from top to bottom: temperature in the close vicinity of the top surface of the crystal; current in the circuit; endpoint energy of the X-ray spectrum; X-ray intensity integrated over 100 s. Blue areas correspond to the cooling phase, red areas correspond to heating phase. Two lower graphs present the X-ray spectra accumulated over a half cycle at negative **(b)** and positive **(c)** polarities. The characteristics X-ray lines are marked in the graphs.

The regime for temperature variation of the LiTaO_3_ crystal is also a key aspect for stable particle generation^15,33^. Smooth temperature variation leads to smooth change of the charge accumulated on a polar surface of the crystal; on the other hand, periodical (sinusoidal) variation of the temperature leads to oscillations of the magnitude and polarity of the accumulated charge^42,43^. We have made the first attempt to generate stable X-rays^44^however the parameters of excitation were not well optimized, which is clearly seen from the current and X-ray graphs. Such regime has previously been used for precise determination of pyroelectric coefficient of the crystal^45,46^. In this paper we applied sinusoidal temperature variation for quasi-continuous X-ray generation and to stabilise the emission optimizing pressure level, crystal-to-target distance and temperature variation rate.

## Results

The key components of the experimental installation as well as two schemes to generate X-ray at positive and negative polarities are illustrated in Fig. 1a. Description of each individual component and their operation is included in the Methods. In every experiment we simultaneously measured.

X-ray spectra, particle current through the circuit, pressure in the vacuum vessel, and temperature in the vicinity of top and bottom surfaces of the LiTaO_3_ crystal, while periodically varying the temperature by the Peltier element attached to the bottom surface.

Figure 2 illustrates the measurement results for temperature variation frequency of 0.5 mHz and for the pressure range from 1.0 to 2.5 mTorr. Due to efficient cooling system we have observed reproducible temperature oscillations at the top surface of the crystal during the entire experiment (more than two hours). Moreover, the current of charged particles and X-ray flow demonstrate periodical stability and reproducibility cycle by cycle.

According to the classical theory of pyroelectricity the current (*I*) of electrons induced by the pyroelectric effect in a closed circuit powered by the crystal^42^

1 $$\:I=\frac{dQ}{dt}=A\gamma\:\frac{dT}{dt},$$

where *Q* is the accumulated charge, *A* is the surface area of the crystal, $\:\gamma\:$ is the pyroelectric coefficient, *T* is the temperature. If the temperature changes according to sinusoidal function as $\:T=\:{T}_{av}\:+\:{T}_{0}\text{s}\text{i}\text{n}\left(2\pi\:ft\right)$ with *f* being the temperature variation frequency, *T*_*av*_ being the average temperature applied, and *T*_*0*_ is the amplitude of temperature variation, the current will change as $\:I=2\pi\:fA\gamma\:{T}_{0}\text{c}\text{o}\text{s}\left(2\pi\:ft\right)$, i.e. the phase of the current is exactly *π*/2 behind the temperature phase.

In Fig. 2a (second graph from top) the current polarity periodically changes. The asymmetry in each half-cycle appears because there is an avalanche process due to impact ionization of the anode material accompanying the characteristic X-ray generation mechanism. The secondary charge multiplication due to ionization is not taken into account in Eq. (1).

The shape can be split into two components: sinusoidal component with amplitude of the order of 100 pA which is described by Eq. (1) and two sharper peaks at positive and negative polarities. The sine current component is shifted by *π*/2 with respect to the temperature phase as predicted by the theory^42^.

The extra peaks in positive and negative polarities appears due to the fact that charged particles ejected from the crystal or target surface (depending on the polarity as shown in Fig. 1b and c) are accelerated in the electric field, bombard the target or crystal surfaces ionizing the atoms and generating additional free charges contributing to the total current. The shift of the peak with respect to the sinusoidal current appears because it takes time to accumulate the electrostatic field to accelerate the electrons to the energy sufficient to initiate impact ionization.

Each X-ray burst in Fig. 2a (fourth graph from the top) coincides with the extra peak in the current plot for both polarities providing an evidence that the ionization process, which is responsible for the characteristic X-ray generation, directly contributes to the current carrier multiplication.

The spectra are different for different polarities. For negative polarity (Fig. 2b) we observe characteristic lines of Copper (K_α_: 8.05 keV) and Zinc (K_α_: 8.64 keV) corresponding to the elements of the brass target, as well as Chromium (K_α_: 5.40 keV) and Iron (K_α_: 6.40 keV) corresponding to the elements of the vacuum chamber. For positive polarity (Fig. 2c) we observe characteristic lines of Tantalum (L_α_: 8.15 keV, L_β_: 9.35 keV, L_γ_: 10.89 keV). The shape of bremsstrahlung spectrum above 20 keV is also different for different polarities: in the case of positive polarity the spectrum decays faster leading to slightly lower endpoint energy than in the case of negative polarity. Theoretically, in rare events one electron might give its almost entire energy to a single bremsstrahlung photon. In other words, the endpoint energy corresponds to the maximal electron energy gained in the pyroelectric accelerator. Therefore, the field configuration generated at positive and negative polarities are different. Nevertheless, the shape of the burst and the X-ray intensity are comparable providing a quasi-continuous and stable X-ray generation.

**Fig. 3:**
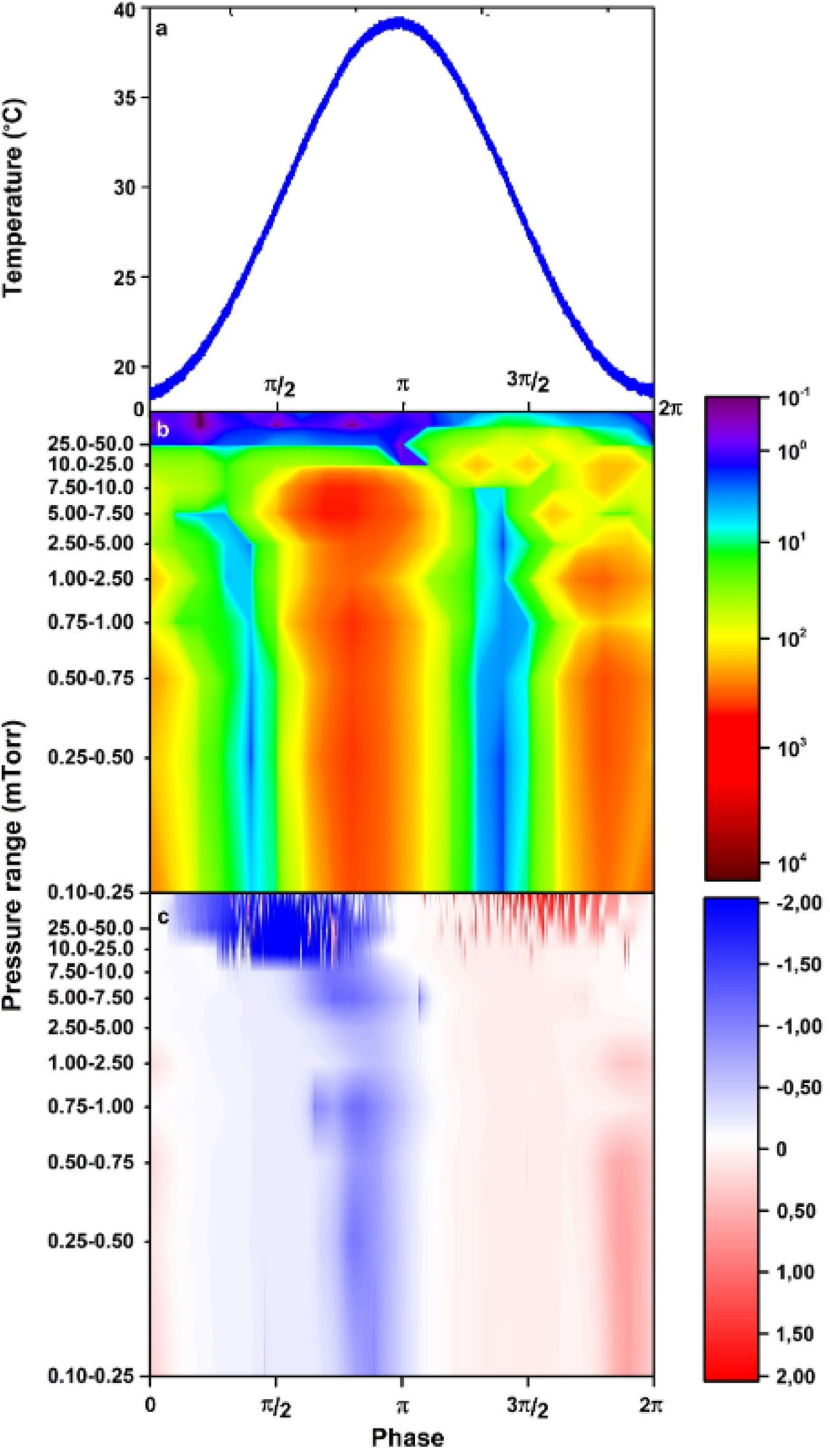
Results of measurements of a single period of temperature variation at 0.5 mHz frequency. From top to bottom we present: **(a)** the temperature variation of the LiTaO_3_ measured at the top surface; **(b)** the map of the total number of counts in the X-ray spectrum averaged over 100 s and in the solid angle captured by the detector (see the Methods); the colour intensity scale is logarithmic; **(c)** the map of the current through pyroelectric accelerator (nA), blue is the negative polarity and red is the positive polarity. The total number of counts (b) and the current (c) are given as functions of the phase and the pressure. Horizontal scale is the temperature variation phase within one thermal cycle. The measurement time was 700 sec.

The stability of the pyroelectric accelerator depends on the pressure level. Figure 3 illustrates the total number of counts in the X-ray spectrum (3b) and corresponding current through pyroelectric accelerator (3c) as a function of different pressure ranges presented for a single period of temperature oscillations (Fig. 3a) at 0.5 mHz. Both regimes of heating and cooling in a single cycle are presented. The maxima of the current are naturally aligned with the maxima of the X-ray bursts. However, in the pressure region in the vicinity of and above 10 mTorr, the current and X-ray generation becomes unstable. This phenomenon is related to the increase of the number of residual gas molecules. As a result, the rate of interaction of charged particles with the gas increases, the total current increases due to impact ionization; however, the X-ray photon count rate decreases because the large number of new particles do not gain sufficient energy to generate X-rays.

One may observe many sharp breakdown spikes in the current map (Fig. 3b) at the pressure level above 10 mTorr. Those spikes are more pronounced at the largest temperature gradients at around *π*/2 and 3*π*/2 phase. Occasionally, they appear even at lower pressure as well (Fig. 2 current plot). According to Eq. (1) the current (as well as the potential in the gap) is the highest at the largest temperature gradient. We believe those spikes appear due to inhomogeneities and defects within the crystal composition and the presence of the residual gas related extra free charges. Nevertheless, the instabilities disappear at the pressure level below 10 mTorr providing a stable and reproducible particle generation regime.

## Discussion

### Characterization of the avalanche discharge

The time evolution of the current induced at the X-ray production in the pyroelectric accelerator has never been investigated in sufficient details so far. Meanwhile, this information can bring light to the understanding of the process between the crystal and the target at thermal excitation of the pyroelectric generator. Previously (see, for example^48,49^and references therein), temperature was instantly applied to one side of the crystal leading to a sharp increase of the current and, subsequent, slow decay during the crystal relaxation time defined by temperature stabilization period. The risetime evolution of the current was too quick to be studied. Moreover, every cycle of the pyroelectric accelerator was different, because the initial crystal temperature was always different. Sinusoidal temperature variation with proper residual temperature extraction allows us to keep the identity of the accelerator operation cycle-by-cycle. On the other hand, slow and smooth temperature change (much slower than the relaxation time) allows us to observe the current evolution in time and characterize the charge behaviour during the pyroelectric accelerator operation.

In Figs. 2 and 3 we have noticed that on top of sinusoidal current induced in the circuit we see extra peaks which are perfectly aligned with the stream of X-rays. Moreover, this peculiarity is observed at positive and negative polarities, i.e. at both directions of the electron flow. The fact that the electrons start generating characteristic X-ray photons means that the energy of electrons is strong enough to initiate impact ionization of the crystal or target atoms (depending on the polarity) and residual gas molecules. Ionization leads to the avalanche grows of the number of free charges acting as current carries and leading to the increase of the current in the circuit. This situation is very similar to a so-called Townsend discharge^50–53^. In this case the avalanche multiplication of electrons between cathode and anode due to impact ionization of the residual gas molecules is observed. However, in our case the residual gas stops affecting the current flow at the pressure level below 10 mTorr.

An avalanche discharge is characterised by a so-called V-I dependence (Fig. 9 in Ref. 52). We measure the current in the circuit directly. The maximal energy of the bremsstrahlung photons (the end point energy) is practically equal to the maximal energy of the electrons accelerated in the gap between the crystal surface and the target. Therefore, the electric field strength can be evaluated as.

 

2 $$\:E=\:\frac{V}{d}\:=\:\frac{\hslash\:{\omega\:}_{max}}{ed}$$

where *V* is the potential in the gap, *d* is the gap size, *e* is the electron charge, and $\:\hslash\:$*ω*_max_ is the maximal X-ray photon energy. The estimation (2) is valid assuming the electric field in the gap is uniform like in a parallel-plate capacitor. The uniformity of the electric field has been studied experimentally^54^. If the endpoint energy of the X-ray spectra changes, the electric field strength in the gap between the crystal and target changes as well.

**Fig. 4:**
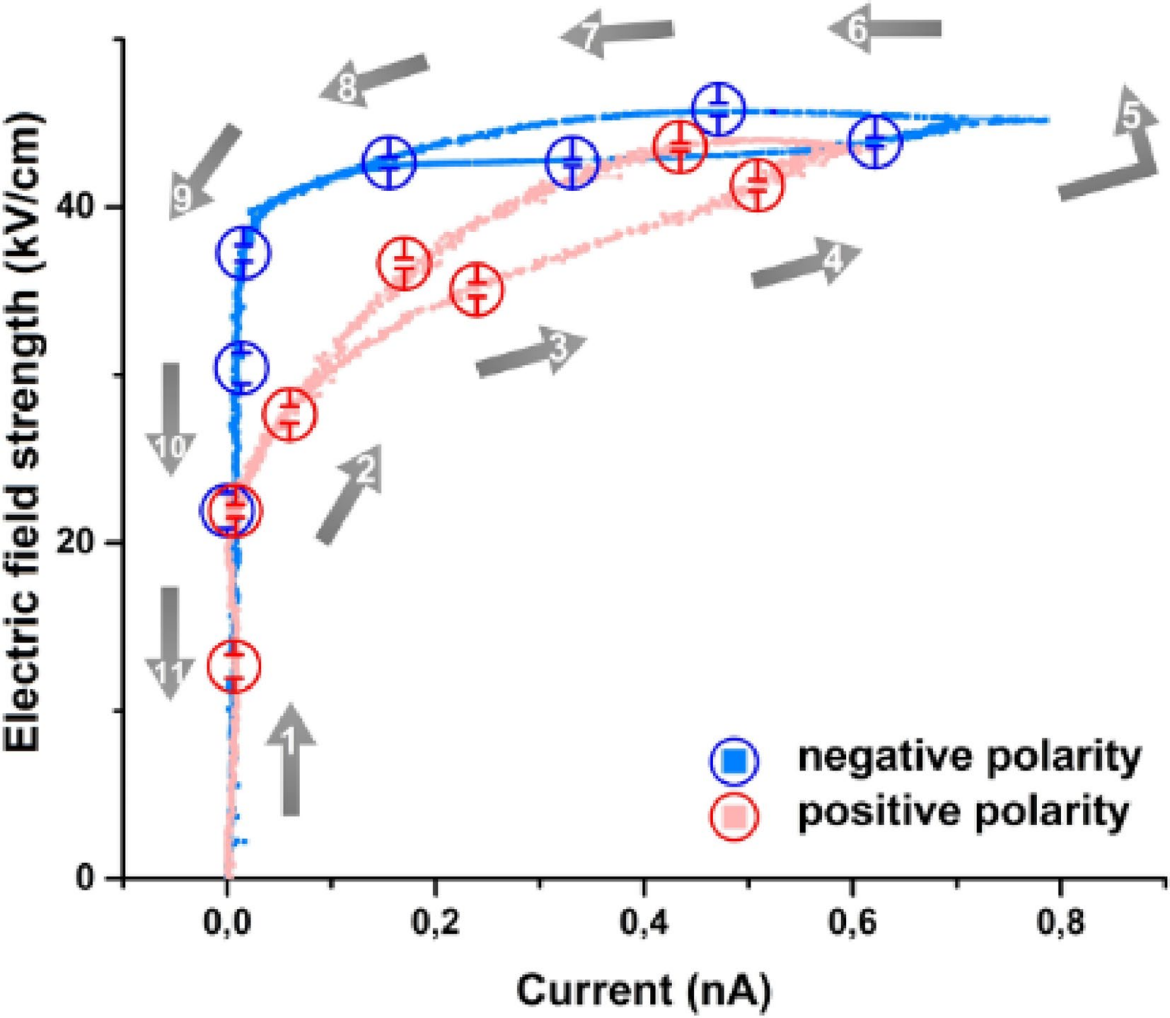
Avalanche process identification – an equivalent of V-I characteristics. Horizontal axis is the current amplitude; vertical axis is the electric field strength in the gap. The red graph presents positive polarity half-cycle, and blue graph presents negative polarity half-cycle. The pressure was in the range from 0.5–0.75 mTorr. The temperature oscillation frequency was 0.5 mHz. The arrows demonstrate the direction of the cycle. The end point energy used to calculate the electric field strength and corresponding to each current point was determined by means of gaussian interpolation and extrapolation of the third temperature cycle shown in Fig. 2. The circle points represent the measured data with uncertainties.

Figure 4 illustrates an equivalent of the V-I dependence characterising the avalanche discharge process in the pyroelectric accelerator system suggesting the following description. Initially, when the temperature starts changing, the potential in the gap between the crystal and the target is growing leading to field electron emission. The number of electrons is growing however the X-rays are not generated within the interval from 0.7 to 72.5 keV, corresponding to the sensitivity of our detector (see “Methods”), because the energy is not high enough. At some point the electrons reach the energy values sufficient to initiate the impact ionization of the anode material. It leads to the increase of charge carriers and appearance of the characteristic and higher energy bremsstrahlung X-ray photons. The current keeps on growing while the endpoint energy saturates. Due to the fact that the temperature gradient reduces, the potential in the crystal-target gap is reduced as well leading to a slow decrease of the potential, and, as a consequence, the endpoint energy and the current in the circuit decrease too. When the current changes its polarity, the process repeats with an inversed direction of the current. The direction of the electron flow is also confirmed by the characteristic X-ray spectra, demonstrating X-rays from the target at negative polarity and from the crystal at positive polarity.

A key characteristic peculiarity of the avalanche process is that the electron energy grows faster than decreases closing the loop in Fig. 4. Moreover, the discharge evolution is different at different polarities because the work function of metal target is smaller than of the crystal^7,55,56^. Therefore, ejections of electrons from the target at positive polarity begins at lower strength of electric field. However, since the total charge generated at positive and negative polarities are comparable, the maximum current at positive polarity is smaller than at negative one.

Figure 4 also demonstrates a small change in the end-point energy in the vicinity of the V-I plot loop confirming the fact that the generated electrons have very small energy deviation. Generation of mono-energetic electrons in a pyroelectric accelerator has already been demonstrated before^1,11–13,22^; however, it has never been properly explained. It is clear that the electron ejection begins when the electric field strength in the gap is strong enough to provide the electrons sufficient energy to surpass the potential barrier. Electrons gain energy in the gap taking out a part of the electric field power. The power is continuously topped up by the varying temperature of the crystal. If the potential is too high, the number of ejected electrons increases keeping the average energy per electron within a narrow energy band.

#### Applicability of the pyroelectric source for energy dispersive X-ray spectroscopy

Nowadays the state of the art in X-ray physics is represented by large central facilities utilizing synchrotron radiation including synchrotron storage rings and linear free electron laser facilities. They cover practically all scientific and technological needs around the world in case if a sample can be brought to the facility for further X-ray processing.

During the past 15 years pyroelectric particle generators were considered as an alternative and economically feasible source for different applications when a mobile source is needed, e.g. for space industry^39^. Our method can be used by geologists in field trips for quick and efficient sample analysis. Light weight and compactness will allow them to take the device along and perform research during a field trip. There were several applications implementing X-ray fluorescent and nano-structural analysis^20,34,35,37–39^. Moreover, mass particles have been studied as an alternative to electron irradiation for medical applications^57^ion mass spectroscopy^9,58^neutrino and dark matter detector calibration^47^. However, these sources have never been used for routine operation because of unstable emission leading to irreproducible results and degradation of the source in time.

There are two key reasons responsible for the obstacles. First of all, the charge relaxation in the pyroelectric crystal is very slow. Therefore, even after a considerably long period of time, new thermal influence begins at a different initial charge state of the crystal leading to a new stream of particles with different intensity and spectral characteristics.

One may degrade the vacuum to slightly reduce the relaxation time; however, this is not acceptable for several applications and, as it is shown in Fig. 3, it leads to an unstable particle emission regime. The second reason is a unique sensitivity of the discussed phenomena to temperature variation dynamics. Small differences at cooling and heating cycles lead to disbalance between positive and negative charge induction at long term thermal variation.

Application of periodically variable temperature eliminates both of these problems. In this case cooling and heating half-cycles are applied in similar manner. Smooth and continuous variation of the temperature change rate enables us to exclude sharp changes of the accumulated charge leading to parasitic electrical breakdowns and unstable particle generation regime. Our research has demonstrated that the more reproducible temperature is cycle-by-cycle, the more stable and predictable the particle generation regime is.

**Fig. 5:**
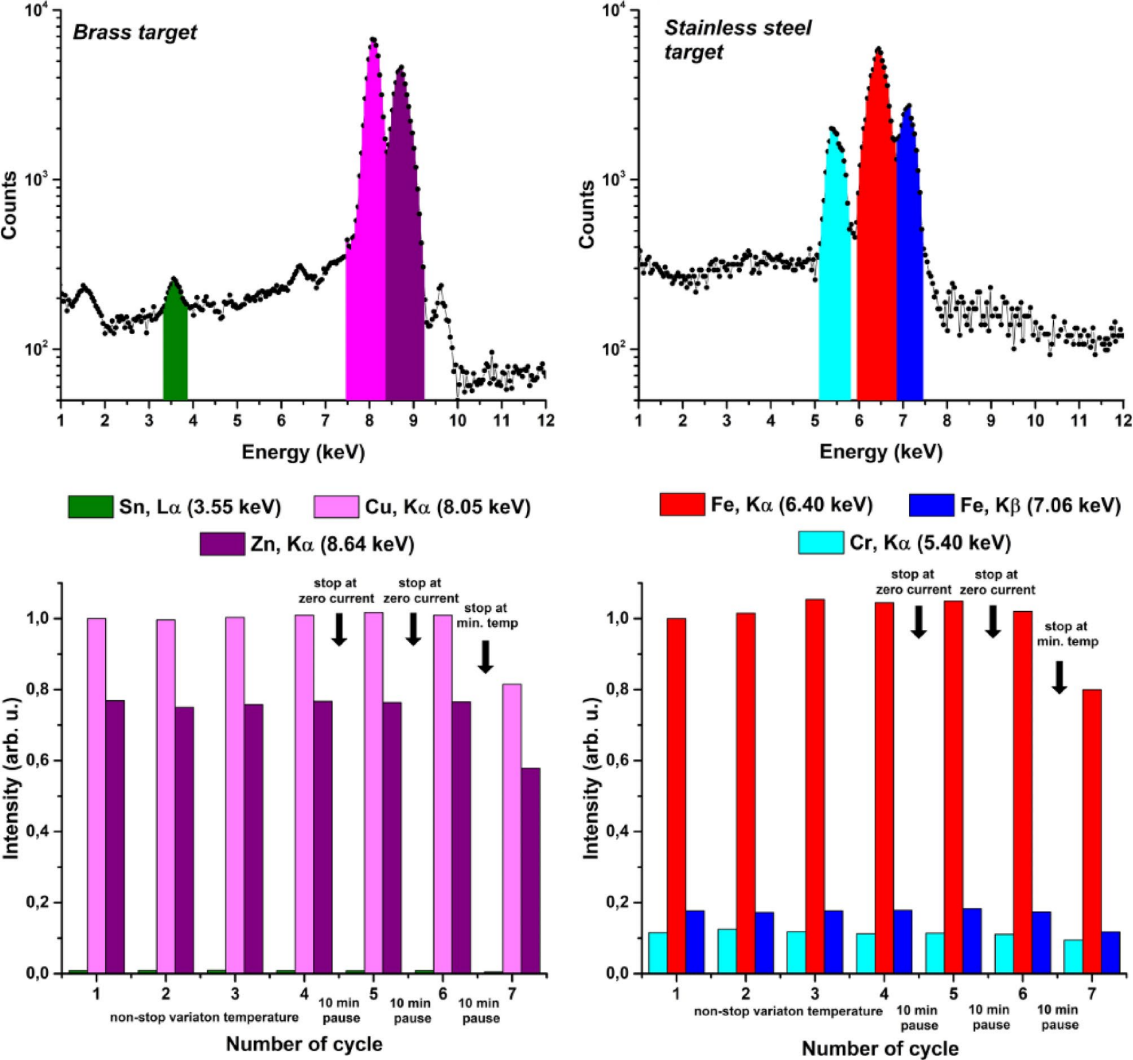
Stability of Energy-Dispersive X-ray Spectroscopy. **(a)** and **(b)** present typical X-ray spectra from brass and stainless steel targets respectively. Marked areas confine photon counts corresponding to characteristic X-rays lines of target elements. **(c)** and **(d)** present a histogram of dynamic change of intensity of highlighted regions in X-ray spectra for brass and stainless-steel targets respectively. The unity in intensity scale is 6.4 ⋅ 10^4^ counts (c) and 6.35 ⋅ 10^4^ counts (d). The horizontal scale represents the thermal cycle number. The fifth and sixth cycles were observed after the measurement were stopped at zero current in the circuit and paused for 10 min. The seventh cycle was observed after the measurements were stopped at minimum temperature and paused for 10 min. The background count during the measurements was a small fraction of a percent.

Here we present an application of an X-ray energy dispersive spectroscopy (EDS) method for element analysis.

using a compact pyroelectric X-ray source. As samples we employed brass and stainless steel. The electron stream generated from the LiTaO_3_ crystal bombarded the samples generating characteristic X-ray lines that provide information about the sample element content. The main emphasis was dedicated to stability and reproducibility of the results. Figure 5 illustrates the analysis of two samples: brass and stainless steel, and demonstrates the stability of the results at long term thermocycling. All key elements were registered. For stainless steel we detected Iron (K_α_ at 6.40 keV) and Chromium (K_α_ at 5.40 keV). Moreover, we detected Nickel (K_α_ at 7.48 keV), which mass fraction is only 8%. For brass we registered Copper (K_α_ at 8.05 keV) and Zinc (K_α_ at 8.64 keV). On top of that we also registered Iron (K_α_ at 6.40 keV), Silicon (K_α_ at 1.78 keV), and Tin (L_α_ at 3.55 keV), despite of the fact that the mass fractions of these elements are 0.2%, 0.3% and 1.0% respectively.

The lower histograms in Fig. 5 (b and c) demonstrate the integral number of photons in the most intense characteristic.

X-ray lines normalised to 6.4 ⋅ 10^4^ counts of the 8.04 keV line for brass (c) and 6.35 ⋅ 10^4^ counts of the 6.4 keV line for stainless steel. At continuous periodical variation of temperature (cycles 1–4, the total operation time is 2.5 h) the deviation of the registered number of photons is less than 5%. It is important to point out that when the temperature changes periodically there is an equilibrium state of the crystal, i.e. when the current in the circuit is zero. Stopping the temperature variation at this moment enables us to restart the measurement later with minimal distortion of the pyroelectric generator operation state. Stopping the measurements between the cycles 4, 5, and 6 and waiting for 10 min did not change the photon rate in the characteristic X-ray spectrum. On the other hand, when stopping the measurement at a different point (e.g. at minimum temperature), the crystal temperature state changes. Restarting the measurements after that leads to degradation of the X-ray count rate.

### Summary

In this research we managed to stabilize the pyroelectric accelerator by applying a periodically varying temperature with frequency of 0.5 mHz and the amplitude of 20 degrees. We have demonstrated a quasi-continuous X-ray emission with reproducible photon yield, endpoint energy and average burst duration. We have identified the avalanche discharge as a mechanism of intense charge multiplication in the pyroelectric accelerator appearing when the electron energy is high enough to initiate intense impact ionization of the target or crystal material. This mechanism plays a key role in understanding of the operation principle of pyroelectric accelerator, which has never been mentioned in all previous investigations. We can identify two stages of particle generation within the pyroelectric accelerator: field emission is the first step, and the avalanche discharge is the second step. We have clearly observed a residual pressure threshold for the second step initiation. All further technical investigations should aim to establish optimal regimes of temperature oscillation frequency, amplitude, and generation geometry depending on the foreseen application of the source.

The EDS method has demonstrated that a mass fraction of 0.1% can be determined with a pyroelectric accelerator system and Cd-Te based spectrometer. A stable, reliable and reproducible result can be achieved. On the other hand, the use of a detector with better spectral resolution (e.g. Si-detector) would provide determination of even lower mass fractions.

The pyroelectric generator has key advantages in comparison with other sources such as X-ray tubes or conventional accelerators. Together with the miniature dimensions and light weight, the pyroelectric accelerator has no external high voltage power supply or radioactive sources integrated. These peculiarities provide an opportunity to create simple, robust, mobile, and safe X-ray operation system for imaging and spectral applications. Even among compact systems as summarised in Table 1, currently available on the market, pyroelectric system has advantages in terms of compactness and safety.

**Table 1:** Small X-ray systems.

	Ours*	COOL-X* [40]	PIXL** [63]	Moxtek** [64]	Tracer-5 [65]
Weight	< 0.5 kg	< 0.5 kg	4.5 kg	0.5 kg	1.9 kg
Size (m^3^)	10^−3^	10^−3^	0.03	0.04	7⋅10^−3^
Power	0.3 W	0.3 W	25 W	10–100 W	4 W
Voltage	9 V	9 V	n/a	< 50 kV	50 kV
Ph.Rate	10^8^ ph/s	10^8^ ph/s	n/a	10^12^ ph/s	n/a
End point energy	60 keV	35 keV	n/a	50 keV	n/a
Dead time	30%	30%	negligible	negligible	negligible
Appl.	Calibr. EDS XRF	Calibr. EDS XRF	Mapping, Material Science	Imaging. EDS XRF	XRF

* The weight includes power source and detector. ** The power source weight is not included here. It is quite heavy, in particular, 50 kV power supply. Calibr. - is the instrumental calibration of X-ray spectrometer systems. Neutron generation is also possible. These sources are considered as an option for calibrating dark matter detectors. EDS - Energy Dispersive Spectroscopy. XRF - X-Ray Fluorescence analysis.

Deeper understanding of the pyroelectric generator enables us to reconsider the use of pyroelectric systems for nuclear research^27,59,60^. An additional bonus related to generation of strong electric fields and particles using electrification effects of solid volume dielectrics^61,62^ is revealed.

The next technological step would be to design a compact assembly, for commercial use. The vacuum chamber presented in Fig. 1 is an experimental station with a great flexibility in terms of observation, temperature extraction, vacuum manipulation, etc.

## Methods

**Experimental layout in** Fig. 1. The pyroelectric crystal assembly (1) consisting of a LiTaO_3_ single crystal with attached aluminium foil, Peltier element (2) separated by a mylar film from the Al foil, and water-cooled radiator was mounted inside the vacuum vessel (4). The water-cooled radiator is necessary to extract residual heat from the Peltier element, which was powered by an amplified sine wave from an arbitrary waveform generator (5). The water was supplied by an external water pump (6). An infrared camera Flir E30 (7) monitored a side surface of the pyroelectric crystal via 0.5 mm infra-red transparent diamond window. The camera enabled contactless measurement of the temperature. Either brass or stainless steel target (9) was mounted on vacuum actuator (8) 11 mm away from the crystal interface. A semiconductor Amptek Cd-Te XR-100T X-ray detector was mounted on a separate side flange such that the radiation from both LiTaO_3_ top surface and the target was measured with the same angular acceptance. The target and the aluminium foil at the bottom of the crystal formed an electrical circuit for measuring the current using picoammeter Keithley 6485 (11).

**LiTaO**_**3**_ **crystal.** As a crystal we used z-cut single crystal of lithium tantalate (LiTaO_3_) being 20 mm long, 20 mm wide, and 10 mm high. The 10 mm height is along the polarization axis.

**Operation principle.** The periodical temperature variation has been provided by an arbitrary waveform generator (TTi ltd., TGA12104). Sinusoidal wave with frequency of 0.5 mHz, voltage of 2 V and current of 0.04 A was amplified to the power of 0.5 W using the current amplifier (Accel Instruments, Accel 302) and powered the Peltier element. The residual gas (air dominated by nitrogen and oxigen) pressure region 0.1–100 mTorr was divided into 12 parts (4 regions per decade). Due to a long measurement time it was difficult to keep a single pressure value. Every measurement has been done using four full temperature cycles. The X-ray spectrum, current in the circuit and temperature from top and bottom surfaces of the crystal have been recorded synchronously. To minimize the residual temperature fluctuations and crystal instabilities the measurements were always stopped at zero current point in the circuit. To maintain the independence between the measurements, a pause of 20 min was applied between the measurement periods. At each pressure interval the measurements have been done at least twice for the entire four-cycle duration to check the reproducibility of the results.

**X-ray spectrometer.** The X-ray spectrum was measured by an X-ray detector Amptek Cd-Te XR-100T and digital pulse processor PX5. The detector was fed into a side port (see (10) in Fig. 1) at a distance 200 mm far from the centre of the vacuum chamber. Moreover, the detector was placed symmetrically with respect to the target and the crystal providing the same observation angle. The solid angle captured by the detector was 6 × 10^−4^ srad. The spectrum range is 0.7–72.5 keV, peaking time of the spectrometer is 3.2 µs, and the operation temperature is 230 K. The spectral resolution is 420 eV at 6.4 keV X-ray photon energy.

**Current measurement.** The current in the circuit was measured by a picoammeter Keithley 6485. The electrical acquisition system included a protection circuit consisting of two diodes 1N3595 and resistance to prevent any damage of the picoammeter damage due to a high voltage breakdown.

**Contactless temperature measurement.** The temperature was monitored by a thermal camera (FLIR E30, see (7) in Fig. 1). The camera was installed at another side viewport levelled with the crystal. The viewport was equipped with a diamond window, which is transparent for IR region.

## Appendix A: comparison of several small X-ray

### Systems

The table in this appendix summarizes a comparison of different technological solution for small X-ray systems to demonstrate the advantage of the pyroelectric accelerator. The COOL-X and our system are both pyroelectric accelerator-based solutions. COOL-X was a commercially available device; however, it has limited operation resource due to vacuum degradation. Nevertheless, the research in this paper will enable stable and reproducible operation of COOL-X system as well. Comparison with other known systems, such as PIXL^63^, Moxtek^64^, Tracer5^65^, shows that the pyroelectric accelerator can provide greater compactness and lower energy consumption than existing active devices and is able to occupy its niche.

## Supplementary Information

Below is the link to the electronic supplementary material.

Supplementary Material 1

## Data Availability

All data generated or analysed during this study are included in this published article [and its supplementary information files].
